# Fluorescence Spectrometric Determination of Drugs Containing **α**-Methylene Sulfone/Sulfonamide Functional Groups Using *N* ^1^-Methylnicotinamide Chloride as a Fluorogenic Agent

**DOI:** 10.1155/2011/840178

**Published:** 2011-05-16

**Authors:** Khaled M. Elokely, Mohamed A. Eldawy, Mohamed A. Elkersh, Tarek F. El-Moselhy

**Affiliations:** Department of Pharmaceutical Chemistry, Faculty of Pharmacy, Tanta University, Tanta 31527, Egypt

## Abstract

A simple spectrofluorometric method has been developed, adapted, and validated for the quantitative estimation of drugs containing *α*-methylene sulfone/sulfonamide functional groups using *N* ^1^-methylnicotinamide chloride (NMNCl) as fluorogenic agent. The proposed method has been applied successfully to the determination of methyl sulfonyl methane (MSM) **(1)**, tinidazole **(2)**, rofecoxib **(3)**, and nimesulide **(4)** in pure forms, laboratory-prepared mixtures, pharmaceutical dosage forms, spiked human plasma samples, and in volunteer's blood. The method showed linearity over concentration ranging from 1 to 150 *μ*g/mL, 10 to 1000 ng/mL, 1 to 1800 ng/mL, and 30 to 2100 ng/mL for standard solutions of **1**, **2**, **3**, and **4**, respectively, and over concentration ranging from 5 to 150 *μ*g/mL, 10 to 1000 ng/mL, 10 to 1700 ng/mL, and 30 to 2350 ng/mL in spiked human plasma samples of **1**, **2**, **3**, and **4**, respectively. The method showed good accuracy, specificity, and precision in both laboratory-prepared mixtures and in spiked human plasma samples. The proposed method is simple, does not need sophisticated instruments, and is suitable for quality control application, bioavailability, and bioequivalency studies. Besides, its detection limits are comparable to other sophisticated chromatographic methods.

## 1. Introduction

Encouraged by the successful application of the NMNCl methodology to the determination of a similar *α*-methylene carbonyl functional group containing drugs, namely, warfarin [1], pentoxifylline, propafenone hydrochloride and acebutolol hydrochloride [2], the almost isosteric *α*-methylene sulfoxide group, such as proton pump inhibitors (PPIs) and the cyclic *α*-methylene carbonyl group, such as ketamine hydrochloride, griseofulvin, and levonorgestrel (unpublished results), we decided to investigate the possibility of extending the application of such methodology to drugs containing the isosteric *α*-methylene sulfone/sulfonamide functional groups, namely, methyl sulfonyl methane (MSM) **(1)**, tinidazole **(2)**, rofecoxib **(3)**, and nimesulide **(4)**. 

Methyl sulfonyl methane (MSM) (**1**) is a relatively new dietary supplement form of sulfur that is found in our living tissues. MSM supports healthy connective tissues like tendons, ligaments, and muscle. Thus, it is important in conditions such as arthritis, muscle pains, and bursitis. MSM should be considered an integral part of any health care practice because of its physiological action, indirect importance, and current/future uses [3–5]. 

In this report, MSM was used as a model compound and was found to react successfully with NMNCl quantitatively yielding a fluorophore. Because there are marketed dosage forms containing MSM, it was decided to apply the developed method for its analysis in its marketed dietary supplement dosage forms in view of the rather elaborate and costly methodologies reported for its analysis in the available literature. The reported methods include Fourier-transform infrared (FT-IR) spectrometry [6], Raman spectroscopy [7], CIMS [8], GC [9], GC with flame ionization detector [10], GC with flame photometric detection [11], capillary GC [12], GC-MS for estimating volatile sulfur compounds [13, 14], solid-phase microextraction-GC-MS [15], dynamic headspace-GC-MS [16], reversed phase HPLC followed by headspace GC-MS [17], and direct thermal desorption prior to GC-MS application [18]. Tinidazole (**2**) is a synthetic antiprotozoal agent. Survey of the available literature reveals several methods for the analysis of **2** active pharmaceutical ingredient (API) and pharmaceutical formulations in the presence of possible impurities and related substances [19, 20]. These methods include fluorometry involving the reduction of its nitro group and subsequent measurement of its emission intensity at 420 nm [21], UV [22], electrochemical method [23], HPTLC [24, 25], LC/MS/MS [26], HPLC [27], and GLC [28]. Further, **2** was analyzed in biological fluids containing its major metabolite using HPLC-MS [29]. 

Rofecoxib (**3**) is a nonsteroidal anti-inflammatory drug (NSAID) introduced in 1998 with lots of fanfare claiming it a selective COX-2 inhibitor with minimal side effects, and **3** was withdrawn in September, 2004 because of safety concerns regarding its untoward cardiovascular side effects leading to several deaths [30, 31].

Different methods are reported for the determination of **3** in the presence of its degradation products and metabolites [32]. These methods include chemometric methods [32], derivative UV [33, 34], HPTLC [35], HPLC after UV photocyclization [36], and LC-MS [37]. 

Nimesulide (**4**) is a sulfonanilide analogue clinically used anti-inflammatory agent; it is not related to conventional NSAIDs, which usually present a carboxyl or hydroxyl functional group [38]. Several methods were described for the determination of **4** in the presence of its compendia-related substances [39] as well as in the presence of its metabolites [40]. These methods include spectrophotometric ones as near-infrared [41], UV techniques [42], fluorometry using egg phosphatidylcholine liposomes [43], and colorimetric methods [44]. Electrochemical methods involve flow amperometry [45] and adsorptive stripping voltammetry [46]. Separation methods involve HPLC [47, 48] and HPLC-MS/MS [49].

Nakamura and Tamura described the application of the reaction of *N* ^1^-methylnicotinamide chloride (NMNCl) to the analysis of various compounds containing *α*-methylene carbonyl groups [50]. Nakamura and Tamura also made qualitative tests on the reaction mechanism and established the cyclized *α*-adduct fluorophore [50]. This reaction was not tested before for compounds containing *α*-methylene groups adjacent to other functional groups.

All previous publications based on the fluorophore produced by reaction with NMNCl described the utility of this reagent to react with drugs containing active methylene *α* to carbonyl functional groups. This paper formulates our continuous effort to extend the utility of NMNCl to determine different classes of drugs containing active methylene *α* to various groups such as cyclic ketone, (ketamine, griseofulvin, and levonorgestrel) sulfoxide (PPIs), and sulfone/sulfonamide (**1–4**) and even to groups that would produce active methylene upon hydration, for example, methyne group, such as levonorgestrel and ethinyl estradiol (unpublished results). 

This paper describes the application and validation of the reaction of NMNCl with some drugs containing *α*-methylene sulfone groups.

## 2. Results and Discussion

**Figure 1:**
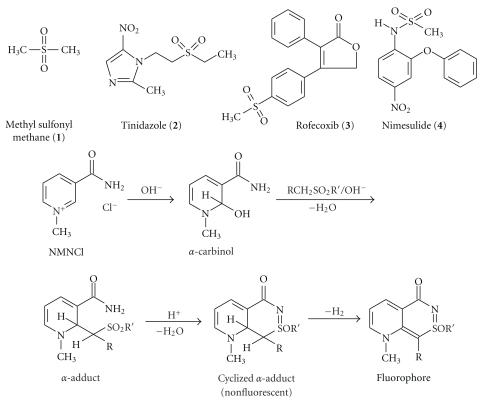
Chemical structures of the analytes and plausible pathway for the reaction of NMNCl with *α*-methylene sulfone/sulfonamide functional groups of **1–4**.

When **1**, **2**, **3**, and **4** (for chemical structures and plausible pathway of the reaction, cf.  Figure 1) were allowed to react with NMNCl under the optimal conditions specified for each, strong fluorescent products were obtained. The optimal wavelengths of excitation and emission of the reaction product were determined using synchronous wavelength search and listed in Table 1.

**Figure 2:**
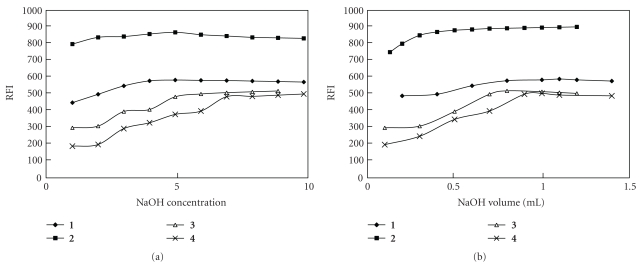
Effect of NaOH concentration and volume on fluorescence intensity of the reaction product of **1–4** with NMNCl. The variation of NaOH concentration is made at constant volume and that of NaOH volume at constant concentration.

**Figure 3:**
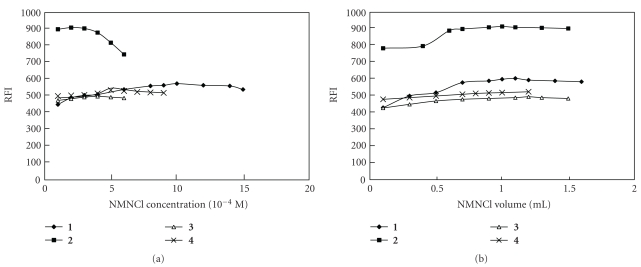
Effect of NMNCl concentration and volume on fluorescence intensity of the reaction product of **1–4** with NMNCl. The variation of NMNCl concentration is made at constant volume and that of NMNCl volume at constant concentration.

**Figure 4:**
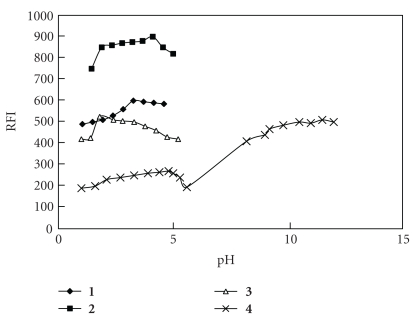
Effect of pH on fluorescence intensity of the reaction and reaction product of **1–4** with NMNCl.

Different variables affecting the reaction between the chosen drugs and NMNCl, including sodium hydroxide concentration and volume, volume and concentration of the added NMNCl, and pH values, were studied to optimize the reaction conditions to give maximum fluorescence intensity (Figures 2, 3, and 4).

Under the optimum conditions for the reaction of NMNCl with the chosen drug, linear relationships between the fluorescence intensity and the drug concentrations were obtained in the following ranges: 1–150 *μ*g/mL, 10–1000 ng/mL, 1–1800 ng/mL, and 30–2100 ng/mL for standard solutions of **1**, **2**, **3**, and **4**, respectively, and over concentration ranges of 5–150 *μ*g/mL, 10–1000 ng/mL, 10–1700 ng/mL, and 30–2350 ng/mL for spiked human plasma samples of **1**, **2**, **3**, and **4**, respectively.

These results have revealed a good and dynamic linearity ranges of the proposed method with different drugs. The good linearity of these relations was indicated by the corresponding regression equations shown in Tables 2 and 3 for standard solutions and spiked human plasma samples, respectively. 

### 2.1. Detection Limit (DL)

Detection limits were practically determined according to the ICH topic Q2B (R1) [51] and found to be 0.5 *μ*g/mL, 3 ng/mL, 0.33 ng/mL, and 10 ng/mL, for standard solutions and 0.7 *μ*g/mL, 5 ng/mL, 0.6 ng/mL, and 18 ng/mL, for plasma samples of **1**, **2**, **3**, and **4**, respectively.

### 2.2. Quantitation Limit (QL)

Quantitation limits were practically determined according to the ICH topic Q2B (R1) [51] and found to be 1 *μ*g/mL, 10 ng/mL, 1 ng/mL, and 30 ng/mL, for standard solutions and 5 *μ*g/mL, 10 ng/mL, 10 ng/mL, and 30 ng/mL, for plasma samples of **1**, **2**, **3**, and **4**, respectively.

### 2.3. Accuracy

The accuracy of the proposed method was studied according to the ICH topic Q2B (R1) [51], by preparing spiked human plasma samples containing various concentrations, lying within the linearity range of each drug, and analyzing them using the proposed method. The results, expressed as % recovery ± S.D., are shown in Table 4 for spiked human plasma samples.

### 2.4. Precision

The precision of the method was judged by performing intraday and interday triplicate analyses of different concentrations covering the linearity range of each drug in spiked human plasma samples. The results are reported as S.D. and coefficient of variation (C.V.) in Table 5 for spiked human plasma samples.

### 2.5. Specificity

To study the specificity of the proposed method, three synthetic mixtures of **1**, **2**, and **3** and two synthetic mixtures of **4** were prepared to contain the possible interfering substances used during pharmaceutical formulations. These mixtures were analyzed using the proposed method and the results, were expressed as % recovery ± S.D., and were as follows: 99.8% ± 3.0 for **1**, 100.3% ± 2.8 for **2**, 99.6% ± 3.5 for **3**, and 100.7% ± 1.7 for **4**.

### 2.6. Assay of Pharmaceutical Preparations

All the pharmaceutical preparations available in the local market for each drug were analyzed using the proposed method. The results, expressed as % recovery ± S.D., are illustrated in Table 6.

### 2.7. Determination of **2** and **4** in Volunteer's Blood

The success in the application of the highly sensitive proposed procedure for the determination of **2** and **4**, in spiked human plasma samples with good accuracy and precision, encouraged the investigator to study its application for monitoring the drug level in the blood of a volunteer receiving **2** or **4** therapy. The level of **2** and **4** was monitored in the blood of volunteers, and their concentrations were found to be 48 *μ*g/mL and 35 *μ*g/mL, respectively, that lie in the therapeutic levels of **2** (47.7 ± 7.5  *μ*g/mL) and **4** (38 ± 10.6 *μ*g/mL).

## 3. Conclusion

The proposed method makes use of the high sensitivity and specificity of the fluorometric analysis to reach low limits of detection and quantitation for all the studied drugs in standard solutions, synthetic mixtures, pharmaceutical preparations, spiked human plasma samples, and patient's or volunteer's blood. The method is simple; it gives results comparable to those obtained by other techniques that require elaborate instrumentation and time-consuming sample preparation procedure.

The method showed good accuracy and precision suitable for quality assurance and could be recommended for bioequivalency and bioavailability studies as well as for validation of cleaning methodology prior to line clearance during manufacture of said dosage forms.

The proposed method application could be extended to cover all available pharmaceutical preparations for each of the chosen drugs.

## 4. Experimental

### 4.1. Apparatus

Shimadzu RF 5301 PC spectrofluorometer.

### 4.2. Materials

#### 4.2.1. Authentic Drugs

**1**, **2**, **3**, and **4** working standards were supplied by Eva Pharma for Pharmaceutical Industries and Medical Appliances, Egypt, Medical Union Pharmaceuticals (MUP), Egypt, October Pharma, Egypt, and Sigma Pharmaceutical Industries, Egypt, respectively.

Plasma samples were purchased from the Central Blood Bank of Tanta University Hospital.

#### 4.2.2. Other Chemicals

*N^1^*-Methylnicotinamide chloride was obtained from Sigma Chemicals Co. Formic acid, sodium hydroxide, methanol, and all other chemicals were of analytical grade. Water used was doubly distilled.

#### 4.2.3. Dosage Forms

*MSM* **(1)**: MSM 1 g tablets (Eva Pharma).

*Tinidazole* **(2)**: Fasigyn 500 mg tablets (Pfizer) and Protozol 500 mg tablets (MUP).

*Rofecoxib* **(3)**: Romacox 25 mg tablets (October Pharma). 

*Nimesulide* **(4)**: Sulide 50 mg and 100 mg tablets (Alkan Pharma), Nimalox 100 mg tablets (Sigma) and sulidan 100 mg tablets (Modern Pharmaceutical Co. (MPC)).

### 4.3. Reagents and Standard Solutions

#### 4.3.1. Stock Standard Solutions of Drugs

Stock standard solutions were prepared in distilled water for **1**, methanol for **3**, and ethanol for **2** and **4** to contain 10 mg/mL, 10 mg/mL, 0.2 mg/mL, and 0.25 mg/mL for **1**, **2**, **3**, and **4**, respectively.

#### 4.3.2. Serial Standard Solutions of Drugs

Aliquots of the stock solution were diluted quantitatively with the same solvent to obtain serial standard solutions in concentration ranging from 0.1 to *15* mg/mL, 0.1 to *10* *μ*g/mL, 0.01 to *20* *μ*g/mL and 0.1 to *250* *μ*g/mL for **1**, **2**, **3**, and **4**, respectively.

### 4.4. Assay Solutions of Drugs in Synthetic Mixtures

Three synthetic mixtures containing **1** along various excipients, additives, and other nonactive ingredients commonly used in pharmaceutical formulations were prepared. The first mixture contained 1000 mg **1**, 135 mg starch, 60 mg gelatin, and 8.0 mg magnesium stearate. The second mixture contained 1000 mg **1**, 75 mg lactose, 30 mg starch, 60 mg gelatin, 8.0 mg magnesium stearate, and 42 mg talc. The third mixture contained 100 mg avicel instead of lactose and gelatin.

Three synthetic mixtures containing **2** were prepared. The first mixture contained 500 mg** 2**, 200 mg cellose, 72 mg starch, 8.0 mg magnesium stearate, 8.0 mg polyethylene glycol and 1.0 mg titanium dioxide. The second mixture contained 500 mg **2** and, 100 mg lactose, 60 mg starch, 60 mg gelatin, 8.0 mg magnesium stearate and 72 mg talc. The third mixture contained 160 mg avicel instead of lactose and gelatin.

Three synthetic mixtures containing **3** were prepared. The first mixture contained 25 mg **3**, 100 mg croscarmellose, 83.5 mg lactose, and 8.0 mg stearate. The second mixture contained 25 mg **3**, 60 mg citric acid, 80 mg sodium citrate, and 10% sorbitol solution. The third mixture contained 25 mg **3**, 235 mg lactose, 60 mg gelatin, 8.0 mg magnesium stearate, and 72 mg talc.

Two synthetic mixtures containing **4** along with various excipients and additives were prepared. The first mixture contained 100 mg **4**, 200 mg lactose, 60 mg starch, 60 mg gelatin, 8.0 mg magnesium stearate, and 72 mg talc. The second mixture contained 260 mg avicel instead of lactose and gelatin.

Each synthetic mixture containing **1**, **2**, **3**, or **4** was extracted with 100 mL of distilled water for **1**, methanol for **2**, or ethanol for **3** and **4**, filtered, and the first 10 mL of the filtrate was rejected. Aliquots of the filtrate were diluted with the same solvents to obtain serial dilutions in concentrations ranging from 0.1 to 25 mg/mL, 0.1 to 10 *μ*g/mL, 0.01 to 20 *μ*g/mL and 0.1 to 25 *μ*g/mL, for **1**, **2**, **3**, and **4**, respectively.

### 4.5. Assay Solutions of Drugs in Their Pharmaceutical Preparations

Twenty tablets were finely powdered, a quantity of the powder, equivalent to one tablet of **1–4**, was transferred with the aid of several portions of distilled water for **1**, methanol for **3** or ethanol for** 2**,** 4 **to a 100 mL volumetric flask and the volume was completed with the same solvent. The resulting solution was filtered and the first 10 mL of the filtrate was rejected. Aliquots of the filtrate were diluted with the same solvents to obtain 100 *μ*g/mL, 6 *μ*g/mL, 5 *μ*g/mL and 6 *μ*g/mL solutions, for **1**,** 2**,** 3** and **4**, respectively.

### 4.6. Assay Solutions of Drugs in Spiked Human Plasma Samples

#### 4.6.1. Serial Standard Solutions of the Drugs

Serial standard solutions were prepared in distilled water (for **1**), in methanol (for **2**), and in ethanol (for **3** and **4**) in concentrations ranging from 1 to 150 mg/mL, 0.01 to 1 mg/mL, 0.001 to 2.0 mg/mL and 0.01 to 2.5 mg/mL of **1**, **2**, **3**, and **4**, respectively.

#### 4.6.2. Preparation of Spiked Human Plasma Samples

Two hundred *μ*L of each of the serial standard solutions of **1** were diluted with 1800 *μ*L human plasma and vortex mixed to obtain concentrations ranging from 0.1 to 15 mg/mL. **2**, **3**, and **4** 200 *μ*L of each drug serial standard solution were evaporated; the residue was dissolved in 1800 *μ*L human plasma and vortex mixed, 200 *μ*L distilled water was added and vortex mixed to obtain 0.001–0.1 mg/mL, 0.0001–0.2 mg/mL, and 0.001–0.25 mg/mL of **2**, **3**, and **4**, respectively.

### 4.7. Preparation of Assay Solutions of Drugs in Plasma Samples

Two hundred *μ*L of spiked human plasma samples (cf. preparation of spiked human plasma samples) were mixed with 1800 *μ*L methanol and centrifuged for 15 minutes to separate the precipitated protein. The clear supernatant was filtered through Millipore filter (0.45 *μ*m) to obtain solutions in concentration range of 0.01–1.5 mg/mL, 0.1–10 *μ*g/mL, 0.01–20 *μ*g/mL, and 0.1–25 *μ*g/mL for **1**,** 2**,** 3**, and **4**, respectively.

### 4.8. Determination of **2** and **4** in Volunteer's Blood

Blood sample was withdrawn in a test tube to which heparin was previously added and dried. The sample was centrifuged to separate plasma and then treated as previously mentioned under preparation of assay solutions of **2** and **4** in plasma samples (cf. preparation of assay solutions of drugs in plasma samples).

### 4.9. N^1^-Methylnicotinamide Chloride Reagent (NMNCl)

Ten mM solution NMNCl reagent was prepared by dissolving 17.262 g NMNCl in one liter of 10^−4^ N HCl. Aliquots of this solution were diluted with distilled water to obtain 1.0 mM, 2.0 × 10^−1^ mM, 4.0 × 10^−1^ mM, and 5.0 × 10^−1^ mM solutions.

### 4.10. General Fluorometric Procedure

One milliliter of each drug standard solutions, assay solutions of synthetic mixtures, assay solutions of pharmaceutical preparations, assay solutions of plasma samples, or the assay solution of the volunteer's plasma was transferred to 10.0 mL screw-capped test tube. Solutions of sodium hydroxide and NMNCl were added. The mixture was cooled (in ice) for the indicated time, then the pH was adjusted using formic acid and heated for the indicated time and then was cooled in ice for 5 minutes (optimum NaOH concentration and volume, volume and concentration of added NMNCl, reaction pH values) and cooling and heating times are indicated in Table 1. The mixture was transferred to 10.0 mL volumetric flask, and the resulting solution was completed using distilled water. In case of **4**, the pH of the reaction product was adjusted to 10.0 before completing to volume with distilled water. The intensity of the resulting fluorescence was measured at the optimal wavelengths indicated in Table 1. The fluorometric measurements were performed against reagent blank experiments. Concentrations of the drugs were calculated from the corresponding calibration graphs prepared simultaneously.

## Figures and Tables

**Table 1:** Optimum conditions for the fluorometric procedure.

Drug	pH*	NaOH conc. (M)	NaOH volume (mL)	NMNCl conc. (mM)	NMNCl volume (mL)	Cooling time (min)	Heating time (min)	*λ*_ex_ (nm)	*λ*_em_ (nm)
**1**	3.0	6.0	1.1	1.0	0.9	9	6	350	395
**2**	3.2	5.0	1.0	2 × 10^−1^	1.0	10	3	336	391
**3**	2.5	7.0	0.9	4 × 10^−1^	1.2	8	5	354	440
**4**	1.5	8.0	1.0	5 × 10^−1^	0.9	7	3	325	375

*The reaction pH.

**Table 2:** Regression analysis parameters for the determination of **1–4** in standard solutions using the proposed method.

Drug	Linearity range	Slope		Intercept		*R* ^2^
		Mean	SE	Mean	SE	
**1**	1–150 *μ*g/mL	4.9890	0.011	105.66	2.15	0.9997
**2**	10–1000 ng/mL	0.8579	0.010	131.84	1.60	0.9998
**3**	1–1800 ng/mL	0.5130	0.003	72.63	1.10	0.9995
**4**	30–2100 ng/mL	0.4155	0.005	113.20	2.78	0.9999

Average of triplicate analyses, 13 data points.

**Table 3:** Regression analysis parameters for the determination of **1–4** in spiked human plasma samples using the proposed method.

Drug	Linearity range	Slope		Intercept		*R* ^2^
		Mean	SE	Mean	SE	
**1**	5–150 *μ*g/mL	4.8849	0.100	118.53	1.55	0.9991
**2**	10–1000 ng/mL	0.7930	0.020	192.15	1.70	0.999
**3**	10–1700 ng/mL	0.5342	0.010	49.64	1.57	0.9989
**4**	30–2350 ng/mL	0.3110	0.003	254.48	1.35	0.9995

Average of triplicate analyses, 13 data points.

**Table 4:** Recovery data of **1–4** in spiked human plasma samples using the proposed method.

Drug	Claimed drug concentration	*Recovered concentration	% Recovery	Mean % recovery ± S.D.	C.V.
**1 **(*μ*g/mL)	5	5.02	100.4%	100.3 ± 2.0	2.0%
	20	20.20	101.0%		
	60	59.00	98.3%		
	80	78.00	97.5%		
	100	102.30	102.3%		
	150	153.00	102.0%		

**2 **(ng/mL)	10	9.8	98.00%	99.98 ± 1.9	1.9%
	30	30.5	101.60%		
	50	51.0	102.00%		
	100	102.0	102.00%		
	300	295.0	98.30%		
	500	490.0	98.00%		
	800	810.0	101.20%		
	1000	987.0	98.70%		

**3 **(ng/mL)	10	10.2	102.0%	100.4 ± 1.4	1.4%
	100	102.0	102.0%		
	300	297.0	99.0%		
	800	807.0	100.9%		
	1000	991.0	99.1%		
	1500	1515.0	101.0%		
	1700	1685.0	99.1%		

**4 **(ng/mL)	30	29.6	98.7%	99.96 ± 1.5	1.5%
	100	98.0	98.0%		
	500	509.0	101.8%		
	1000	1015.0	101.0%		
	1200	1181.0	98.4%		
	1500	152.5	101.6%		
	1800	1809.0	100.5%		
	2100	2345.0	99.7%		

*Average of triplicate analyses.

**Table 5:** Intraday and interday precision of **1–4** determination in plasma samples using the proposed method.

Drug	Claimed conc.		Intraday			Interday	
		Found conc*	S.D.	C.V.	Found conc*	S.D.	C.V.
**1 **(*μ*g/mL)	5	5.03	0.02	0.4%	4.9	0.71	0.14%
	20	20.1	0.07	0.4%	19.7	0.21	1.10%
	60	61.3	0.90	1.5%	58.6	0.99	1.70%
	80	78.0	0.14	1.7%	83.0	2.12	2.60%
	100	104.0	2.80	2.8%	97.0	2.12	2.15%
	150	154.0	2.80	1.9%	146.0	2.80	1.90%

**2 **(ng/mL)	10	9.7	0.21	2.2%	9.8	0.14	1.42%
	30	29.3	0.50	1.7%	30.5	0.35	1.16%
	50	51.5	1.10	2.1%	49.0	0.71	1.43%
	100	102.0	1.40	1.4%	102.0	1.40	1.40%
	300	296.0	2.80	1.0%	289.0	7.78	2.60%
	500	512.0	8.40	1.7%	510.0	7.10	1.40%
	800	795.0	3.50	0.4%	810.0	7.10	1.87%
	1000	985.0	10.60	1.1%	985.0	10.60	1.10%

**3 **(ng/mL)	10	10.4	0.30	2.8%	10.3	0.21	2.08%
	100	96.0	2.80	2.9%	96.0	2.82	2.90%
	300	304.0	2.80	0.9%	305.0	3.50	1.20%
	500	495.0	0.35	0.7%	506.0	4.24	0.84%
	1000	1020.0	14.10	1.4%	1020.0	14.10	1.40%
	1100	1125.0	17.70	1.6%	1125.0	17.70	1.60%
	1500	1475.0	17.70	1.2%	1485.0	10.60	0.71%
	1700	1720.0	14.10	0.8%	1720.0	14.10	0.83%

**4 **(ng/mL)	30	29.4	0.40	1.40%	30.3	0.21	0.70%
	100	98.0	1.40	1.40%	104.0	2.83	2.80%
	500	505.0	3.50	0.70%	491.0	6.36	1.28%
	1000	1015.0	10.60	1.10%	980.0	14.10	1.40%
	1200	1185.0	10.60	0.90%	1225.0	17.70	1.45%
	1500	1524.0	16.90	1.10%	1529.0	20.50	1.35%
	1800	1815.0	10.60	0.60%	1780.0	14.10	0.79%
	2350	2340.0	7.10	0.30%	2360.0	7.10	0.30%

*Average of triplicate analyses.

**Table 6:** Results of the recovery experiments of **1–4** in different pharmaceutical preparations.

Drug	Pharmaceutical preparations	% Recovery ± SD*
**1**	(**MSM**) 1000 mg tablet	101 ± 2.0%

**2**	(**Protozol**) 500 mg tablet	101 ± 1.6%
	(**Fasigyn**) 500 mg tablet	98 ± 2.5%

**3**	(**Romacox**) 25 mg tablet	103 ± 0.7%

**4**	(**Nimalox**) 100 mg tablet	102.2 ± 3.0%
	(**Sulide**) 100 mg tablet	101.6 ± 3.6%
	(**Sulide**) 50 mg tablet	99.9 ± 1.8%
	(**Sulidan**) 100 mg tablet	101.6 ± 3.2%

*Average of triplicate analyses.
